# Maltitol-Derived Sacrificial Agent for Enhancing the Compatibility between PCE and Cement Paste

**DOI:** 10.3390/ma16247515

**Published:** 2023-12-05

**Authors:** Huan Wang, Weixun Zhao, Siqi Wang, Chao Wang, Qifei Du, Yan Yan, Xianke Yang, Sa Lv, Hongliang Hu, Yujie Jin, Lingwei Kong, Ping Wang, Yaodan Chi, Xiaotian Yang

**Affiliations:** 1 Key Laboratory for Comprehensive Energy Saving of Cold Regions Architecture, Ministry of Education, Jilin Jianzhu University, Changchun 130118, China; wanghuan@jlju.edu.cn (H.W.); lvsa82@163.com (S.L.); 2 Department of Materials Science, Jilin Jianzhu University, Changchun 130118, China; 3 Department of Traffic Engineering, Jilin Jianzhu University, Changchun 130118, China; 4 Department of Chemistry, Jilin Normal University, Siping 136000, China

**Keywords:** sacrificial agent, polycarboxylate ether, clays, interactions

## Abstract

At present, it is known that when there is clay in concrete, polycarboxylates (PCE) will preferably adsorb in the clay, so that PCE cannot be fully combined with cement particles, which reduces the workability of the cement slurry. In this paper, a new type of maltitol–ammonium salt cationic (KN-lm) sacrificial agent (SA) has been successfully developed via a simple method, which makes PCE easier to bond with cement particles in the cement slurry containing clay. The effect of KN-lm on the fluidity of clay-containing cement paste is studied, and the experimental results show that KN-lm, as an efficient SA of cement slurry, makes PCE more compatible with clay-containing cement slurry, and increases the initial fluidity of cement slurry by about 19%. Further investigations of TOC, XRD, and zeta potential measurements reveal that a KN-lm ion is only preferably adsorbed into clay compared to PCE through electrostatic adsorption but without having any crystal structure change, thus resulting in good dispersion of cement particles. The addition of KN-lm plays an important role in hindering the hydration expansion of the clay by preferential electrostatic adsorption, which means PCE cannot easily insert into the interlayer of the clay.

## 1. Introduction

Cement-based concrete materials constitute the most extensively utilized type of building materials [1]. Water-reducing agents can improve the flow of fresh concrete without increasing water consumption [2,3]. In the manufacturing process of high-performance cement-based materials, polycarboxylate ether (PCE) is renowned for its widespread application as a superplasticizer due to its low dosage and superior efficiency [4,5], but it does not easily exclude the negative effect of clay originating from sandstone in the concrete [6,7]. This is attributed to the reason that PCE and clay surfaces can absorb a large amount of Ca^2+^, cation electrostatic interaction, and part of the polarized oxygen atoms in the side chain of PCE can bind with water molecules anchored by silanol groups in the aluminum silicate layer [8]; then, the side chain enters the interlayer and forms intercalation, so that the ability of clay minerals to absorb a substantial amount of PCE [9,10] greatly limits the effectiveness of the water-reducing action of PCE cement. This phenomenon, known as poor clay tolerance, significantly hampers the practical application of PCE in cementitious composites that contain clay [11]. In addition, due to the cost and plasticization issues, this problem cannot be solved simply by increasing the dose of PCE. Current efforts to address this have focused on two aspects: (1) modifying the molecular structure of PCE to achieve intercalation adsorption reduction between PCE and clay; (2) introducing an additive that can specifically adsorb clay and inhibit the adsorption of PCE with clay is known as the SA [12]. Plank et al. report that the utilization of polyethylene glycol (PEG) can serve as an effective surfactant when employing highly grafted PCE. Notably, the inclusion of PEG can significantly impede the penetration of PCE into clay, thereby augmenting the clay tolerance of PCE. [13,14]. Dan et al. successfully synthesized 1-tetracyl-3-methylimidazole ammonium bromide ionic liquid as an SA, which also significantly improved the compatibility of PCE with clay-containing cement slurry. However, the preparation process of ionic liquid is complicated, the material cost is high, and it is not suitable for large-scale applications [15]. Zhao et al. used β-cyclodextrin and 3-chloro-2-hydroxypropyl trimethylammonium chloride as raw materials to prepare an SA. Cyclodextrin materials have a high cost and are also not suitable for mass production [16,17]. Hence, developing a low-cost, easily prepared, and mass-produced SA of clay is still highly required.

Here, in this work, a biological SA KN-lm was designed and synthesized. Montmorillonite (Mt) was used as a clay mineral to study the competitive adsorption of Mt by PCE and SA. The feasibility of KN-lm as an SA was determined by the “micro-slump test”. The adsorption mechanism of Mt by KN-lm was studied by TOC, zeta potential, and XRD. The dispersion and microstructure of Mt were characterized by sedimentation and scanning electron microscopy (SEM) [18,19].

## 2. Experimental

### 2.1. Materials

3-chloro-2-hydroxypropyl trimethylammonium chloride (CHPTAC, 65 wt%) was purchased from Shanghai Maclin Biochemical Technology Co., Ltd. (Shanghai, China), maltitol (75 wt%) was purchased from Shandong Yusuo Chemical Technology Co., Ltd. (Jinan, China), sodium hydroxide (99%) purchased from Xilong Science Co., Ltd. (Guangdong, China), hydrochloric acid (99%) purchased from Tianjin Damao Chemical Reagent Factory (Tianjin, China), and anhydrous ethanol (99%) was purchased from Tianjin Xinbote Chemical Co., Ltd. (Tianjin, China).

Cement that satisfies the requirements of BS EN197-1:2011 for CEM II/B-V Portland cement of strength class 42.5 N was used in this study. The commercially available Montmorillonite (Mt) purchased from Shandong Yoso Chemical Technology Co., Ltd. (Jinan, China) was used. The chemical compositions of cement and Mt analyzed are listed in Table 1.

### 2.2. Synthesis of KN-lm

An amount of 4.8 g sodium hydroxide (NaOH) was added to 100 mL of a three-mouth round-bottomed flask containing 15 mL deionized water, and a certain amount of maltitol was added under stirring conditions. The heating temperature was stabilized at 60 °C, and 5 mL of deionized water and a certain amount of 3-chloro-2-hydroxypropyl trimethylammonium chloride (CHPTAC, 65 wt%) of the mixed aqueous solution were uniformly added to the alkali solution containing maltitol under agitation for about 1 h, and the reaction continued at 60 °C for 5 h after the completion of the drip. At the end of the reaction, the pH value of the reaction liquid was adjusted to 8~9 with concentrated hydrochloric acid, and then the clarified reaction droplets were added to a large amount of anhydrous ethanol to form a white precipitate, which was filtered and dried in a vacuum to obtain the white powder, dissolved in deionized water, and again added to anhydrous ethanol to precipitate, repeated three times to obtain the product maltitol-based small molecule clay inhibitor (KN-lm).

The molar ratio of maltitol and 3-chloro-2-hydroxypropyl trimethylammonium chloride (CHPTAC, 65 wt%) was 1:4; 1:6; 1:8; 1:10, respectively.

### 2.3. Test Methods

#### 2.3.1. Mini Slump Test

The evaluation of KN-lm as clay-specific SA of PCE in cement-Mt was conducted by testing the fluidity of cement paste. The optimum synthesis ratio of KN-lm and the optimum KN-lm content were obtained. For the fluidity test of the cement paste, the following is conducted: cement with a water–cement ratio of 0.29 is used as a reference. Add 87 g water to 300 g cement, and add PCE of 0.3% cement mass (0.9 g) under this water–cement ratio. On this basis, KN-lm with different proportions of synthesis was added to it. First, the pulp cleaner was used to stir at a slow speed for 2 min, then stood for 15 s, and then the pulp mixer was used to stir rapidly in reverse for 2 min. Immediately after mixing, pour the cement paste into a clean mold (height 40 mm, top diameter 60 mm, bottom diameter 70 mm) placed on the glass plate, and lift the clean mold vertically. The spreading of cement paste is evaluated in both horizontal and vertical directions, and subsequently, the average is calculated to arrive at the ultimate diffusion value [20].

#### 2.3.2. TOC

For the total organic carbon analysis (TOC) test, by testing the total organic carbon content of Mt before and after adding KN-lm, to analyze the mutual adsorption behavior on the Mt surface, and to determine whether KN-lm can better adsorb Mt and improve the clay tolerance of PCE, proceed as follows: Weigh it at 3 g Mt, add an appropriate amount of water-reducing agent and SA (diluted to 87 g with water), stir with the magnetic force for 4 min, centrifuge treatment (10,000 r/min, 2 min) to absorb the supernatant, and dilute the supernatant by 10 times. The total organic carbon adsorption of Mt and Mt-supernatant with different dosages KN-lm was measured by a TOC total organic carbon analyzer (Shimadsu TOC-VWP, Shimadsu Ltd., Suzhou, China). At the same time, the total organic carbon content of blank contrast samples was measured.

#### 2.3.3. XRD

A quantity of Mt, KN-lm, PCE, and water was added to a glass bottle, which was then subjected to mechanical stirring for 2 min, the suspension was transferred to a centrifugal tube and centrifuged at 9000 rpm for 10 min, the upper layer was removed, and the sediment was obtained by repeated washing with deionized water three times. The sediment was dried in a vacuum at 60 °C for 12 h and then ground to obtain a sample for XRD detection. XRD tests were conducted on the Rigaku UItima type IV X-ray (Rigaku Corporation Ltd., Tokyo, Japan) diffractometer (XRD) with a scanning range of 3~15° and a scanning speed of 1°/min [21].

#### 2.3.4. Zeta Potential

The Zeta potential test can test Mt’s potential and the potential change after adding KN-lm, as follows: weigh 3 g Mt, add appropriate KN-lm (diluted to 87 g with water) [22], stir magnetically for 4 min, centrifuge treatment (10,000 r/min, 2 min) to absorb the supernatant, adjust the pH value with potassium hydroxide buffer solution to 12.0, and then use Zeta potential tester (Zetasizer NanoZS, Malvern Panalytical Ltd., Malvern, UK). Each experiment was repeated five times, and the average value was taken [23,24].

#### 2.3.5. Dispersibility of Mt with KN-lm in Water

Mt and water are added to each of the four glass bottles, resulting in four identical blank suspensions. Subsequently, PCE was added to the second sample bottle, KN-lm was added to the third sample bottle, and PCE and KN-lm were added to the fourth sample bottle. All sample bottles were stirred for 2 min, observed, and recorded for 0 min, 15 min, 30 min, and 60 min, respectively. Dispersion of liquid in four glass bottles [8,25].

#### 2.3.6. SEM

The suspensions of Mt, Mt-PCE, Mt-KN-lm, and Mt-KN-lm-PCE were respectively poured into a centrifuge tube, centrifuged at 9000 rpm for 10 min, rinsed with deionized water three times, and dried at 60 °C for 24 h under vacuum. The obtained solids were ground into powder, and the specimens were characterized by scanning electron microscopy (JSM-7610F, JEOL Ltd., Beijing, China) [26,27].

## 3. Results

### 3.1. Effect of KN-lm on Clay

#### 3.1.1. Influence of Different Synthetic Ratios on the Fluidity of Slurry

##### Containing Montmorillonite

**Figure 1:**
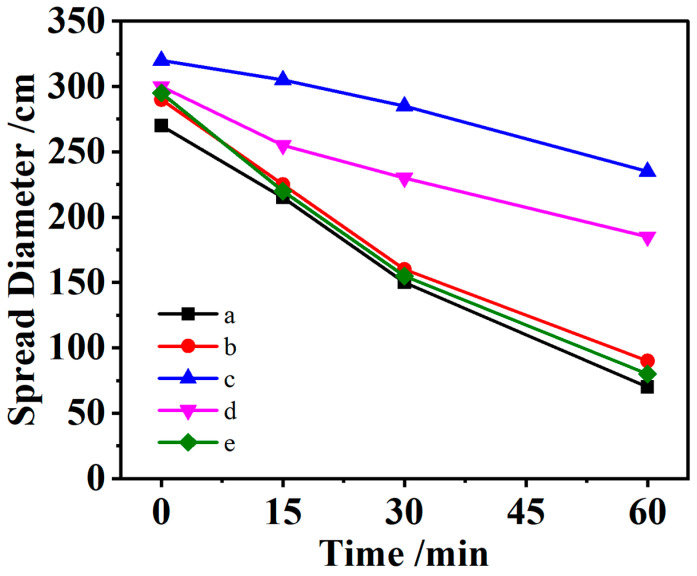
Fluidity of cement slurry synthesized with KN-lm at different ratios of CHPTAC and maltitol (the content of KN-lm was 0.12 wt% of cement mass), respectively. a: Without KN-lm (black square); b: the synthesis ratio is 1:4; c: the synthesis ratio is 1:6; d: the synthesis ratio is 1:8; e: the synthesis ratio is 1:10.

Figure 1 presents the effect of KN-lm with various synthetic ratios on the fluidity of the Mt slurry. The initial fluidity of the cement paste in the blank control group without KN-lm is 270 mm, and when the synthetic molar ratio of 3-chloro-2-hydroxypropyl trimethylammonium chloride to maltitol reaches 1:6, the fluidity of the cement paste is the highest, reaching 320 mm, which is 19% higher than that in the blank group. The fluidity of the cement paste reached 305 mm during 15 min menstruation, and that of the blank control group without KN-lm was 215 mm during 15 min menstruation, an increase of 42%, and that of the cement paste reached 285 mm during 30 min menstruation. In the control group without KN-lm, the flow of cement paste at 30 min was 150 mm, an increase of 90%; in the 60 min control group, the flow of cement paste reached 235 mm; in the blank control group without KN-lm, the flow of cement paste at 60 min is 70 mm, an increase of 236%. It can be seen that KN-lm as an SA can effectively improve the fluidity of clay-containing cement slurry.

#### 3.1.2. Influence of Different Dosages on Fluidity of Slurry Containing Montmorillonite

**Figure 2:**
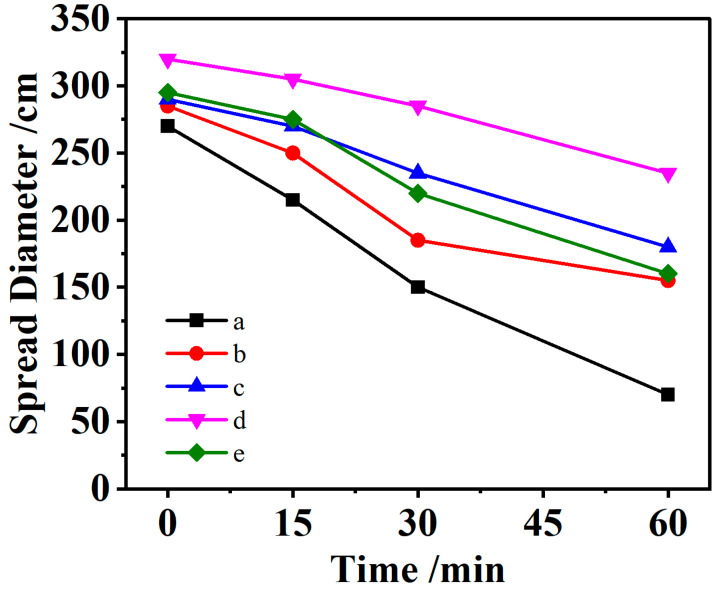
The flow of cement slurry (CHPTAC:Maltitol = 1:6) with different KN-lm contents, a: 0.0‰; b: 0.6‰; c: 0.9‰; d: 1.2‰; e: 1.5‰.

KN-lm exhibits the best mud resistance when the mix ratio is 1:6. Therefore, we studied the influence of different contents of SAs on the fluidity of slurry containing Mt when the mix ratio is 1:6. Figure 2 shows the warp flow variation curve of KN-lm content of 0 wt%, 0.06 wt%, 0.09 wt%, 0.12 wt%, and 0.15 wt%. It can be seen from the results that when the content of KN-lm is 0.12 wt% in cement, the flow of the cement paste exhibits the highest value, and the initial flow of the cement paste reaches 320 mm. In the blank control group without KN-lm, the initial flow of the cement paste is 270 mm, an increase of 19%, and the flow of the cement paste reaches 305 mm after 15 min. In the blank control group without KN-lm, the flow of cement paste at 15 min is 215 mm, an increase of 42%; in the 30 min control group, the flow of cement paste reaches 285 mm; in the blank control group without KN-lm, the flow of cement paste at 30 min is 150 mm, an increase of 90%. The fluidity of the cement paste reached 235 mm at 30 min, and that of the blank control group without KN-lm was 70 mm at 60 min, an increase of 236%.

It should be noted the fluidity of cement paste containing claying after introducing KN-lm is comparable to that with other types of SA. As shown in Table 2; however, our developed KN-lm possesses a lower cost demonstrating its superiority.

### 3.2. TOC

**Figure 3:**
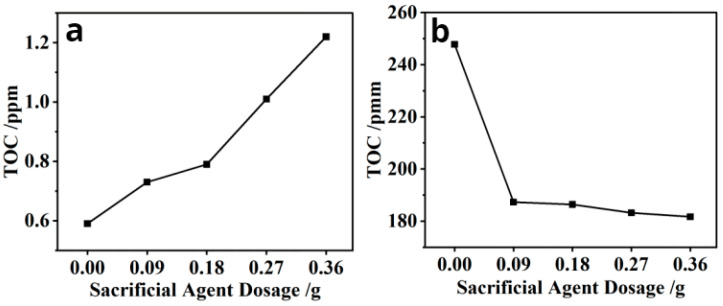
(**a**) The change of total TOC on Mt with the increase of KN-lm dose. (**b**) The change of total TOC on Mt-PCE with the increase of KN-lm dose.

To study the adsorption behavior of PCE on clay in the presence of SA, the TOC technique was used to test the adsorption of a mud repellent on Mt and the adsorption of a mud repellent and water reducer on Mt. Figure 3 shows the total organic carbon adsorption curves of Mt-KN-lm (Figure 3a) and Mt-PCE-KN-lm (Figure 3b), respectively.

As can be seen from Figure 3a, with the increase of KN-lm content, the TOC adsorption capacity of the supernatant also increases, indicating that KN-lm is successfully adsorbed on Mt. As shown in Figure 3b, with the increase of KN-lm content in the Mt-PCE system, the TOC adsorption capacity is significantly decreased, demonstrating that the increase of KN-lm content could induce the decrease of PCE adsorption on Mt, which supports the conclusion that KN-lm can preferentially adsorb onto Mt to improve the clay tolerance of PCE.

### 3.3. XRD

**Figure 4:**
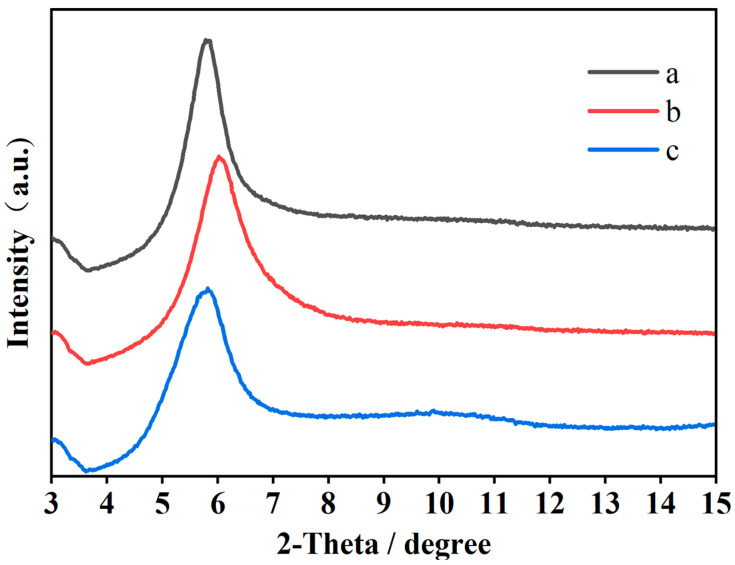
The XRD patterns of Mt (a), Mt-KN-lm (b), and Mt-KN-lm-PCE (c), respectively.

To further study the adsorption mechanism of PCE and Mt in the presence of SA, the XRD of three samples of Mt, Mt-KN-lm, and Mt-KN-lm-PCE were measured, and the results are shown in Figure 4. Compared to Mt, it can be observed that when KN-lm is added the peaks of the Mt-KN-lm and Mt-KN-lm-PCE samples do not possess a significant shift, which reflects that the d value of Mt was not alerted after the introduction of KN-lm. If there exists any intercalation induced by the addition of KN-lm, the peak should exhibit the characteristic of blueshift [18]. This indicates the adsorption of KN-lm on Mt can significantly hinder the insertion of PCE into Mt, and improve the clay tolerance of PCE [8,28].

### 3.4. Zeta

**Figure 5:**
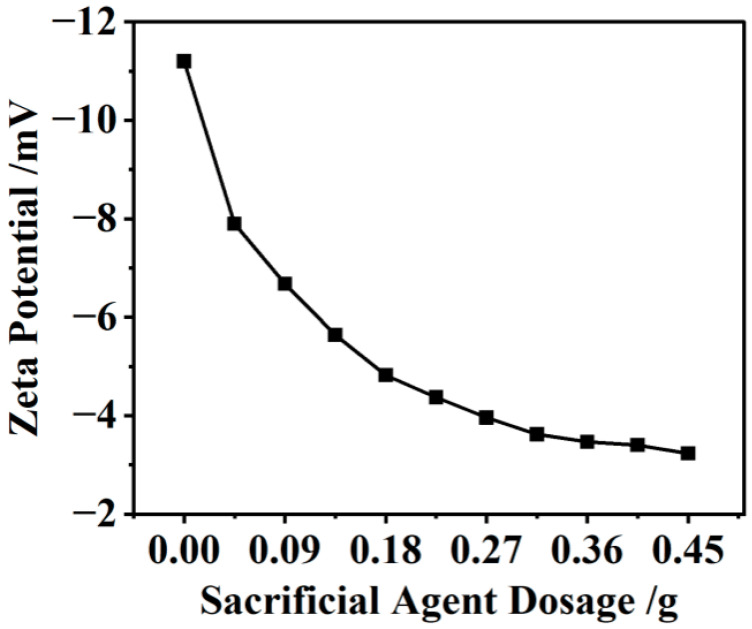
Zeta potential of Mt-KN-lm centrifugal supernatant at pH 12.0 with different KN-lm dosages.

To determine the electrostatic adsorption mechanism of KN-lm and Mt, we further studied the interaction between KN-lm and Mt by observing the change of the zeta potential value. As shown in Figure 5, the zeta potential value of the Mt aqueous solution is −11.2 mV. However, interestingly, with the increase in the content of SA in the corresponding solution, the potential is gradually increased to ~−3.0 mV. In addition, it is known that KN-lm itself contains a positive charge of quaternary ammonium salt; therefore, we rationally propose KN-lm would effectively adsorb onto the surface of Mt, and thus the resulting Mt-KN-lm aqueous solution is unstable owing to the weakened electrostatic repulsion. The variation of zeta potential with KN-lm indicates that KN-lm tends to be adsorbed on Mt.

### 3.5. Dispersibility of Mt with KN-lm in Water

**Figure 6:**
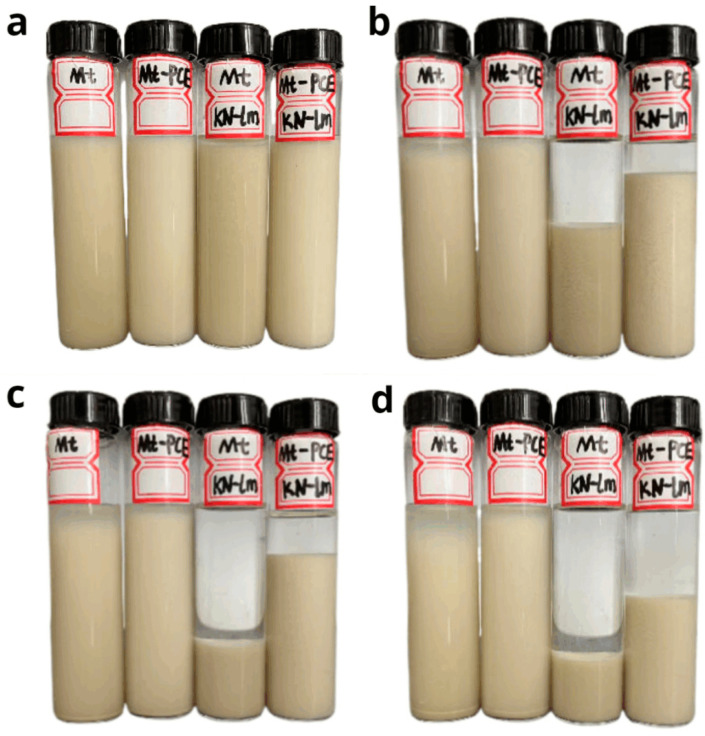
Dispersibility of Mt with KN-lm in water under different standing times (the content of Mt, PCE, KN-lm is 1.5 g, 0.45 g, 0.18 g, respectively, and the water is 43.5 g) (From left to right, Mt, Mt-PCE, Mt-KN-lm, and Mt-PCE-KN-lm), (**a**) 0 min; (**b**) 15 min; (**c**) 30 min; (**d**) 60 min.

The dispersibility measurements of four different cases (Mt, Mt-PCE, Mt-KN-lm, and Mt-PCE-KN-lm) are shown in Figure 6. As can be seen, at the beginning, all the solutions exhibit a homogeneous dispersion. As the incubation time increase, the dispersibility of the one with KN-lm (Mt-KN-lm, and Mt-PCE-KN-lm) is significantly decreased, while for the cases of Mt and Mt-PCE, the corresponding solutions keep their dispersibility well, which supports well that KN-lm is more easily adsorbed onto Mt [8,29].

### 3.6. Scanning Electron Microscopy (SEM)

**Figure 7:**
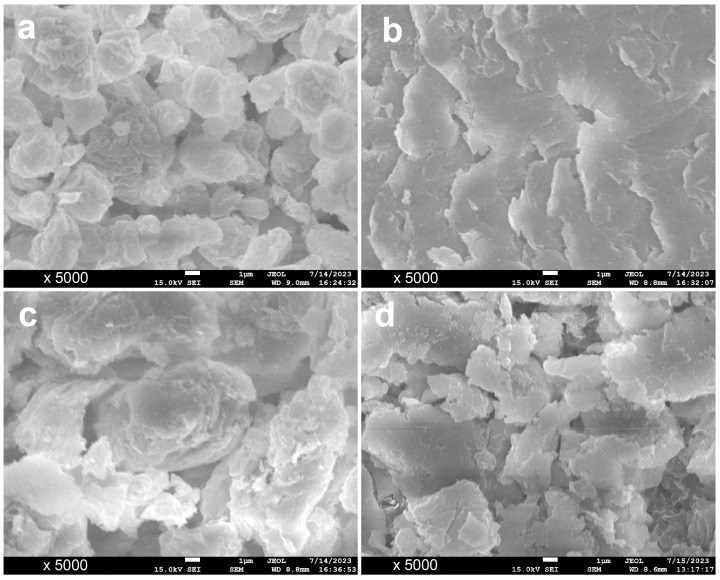
SEM images of Mt (**a**), Mt-KN-lm (**b**), Mt-PCE (**c**), and Mt-PCE-KN-lm (**d**), respectively.

Previous analysis confirms that the dispersibility of Mt-KN-lm aqueous solution is poor compared to that of Mt. SEM was used to investigate the morphology change of different cases. As shown in Figure 7, Mt is only composed of particles of a small size from 0.2 to 1 mm, while Mt-KN-lm (Figure 7b) has a large sheet agglomerate structure; we suppose that a large number of KN-lm may be adsorbed by Mt to form a large-size sheet agglomerate structure, which is similar to the dispersion of Mt-KN-lm in water, further confirming why Mt-KN-lm aqueous solution possesses bad dispersibility [8,30]. Figure 7c is the SEM photo of Mt-PCE, from which it can be found that the morphology of Mt has undergone significant changes and the size is increased, which may be caused by the adsorption of PCE on Mt. Compared to Figure 7c,d, it is found that after the addition of KN-lm SA, the overall particle structure becomes a flake structure, indicating that KN-lm is preferentially adsorbed on Mt, so that PCE and Mt are no longer combined into the particle structure of Figure 7c, and the clay resistance of PCE is improved [31,32].

These results show that KN-lm can be preferentially absorbed onto Mt, and this interaction can well protect PCE from intercalation, therefore restoring the water-reducing effect of PCE, and improves the clay tolerance of PCE in cement-based materials. According to the zeta potential and XRD analysis, the interaction between KN-lm and Mt is mainly due to the surface electrostatic adsorption, but not the intercalation of Mt by KN-lm. It is speculated that KN-lm can prevent Mt hydration and inhibit clay expansion, and PCE cannot reach the interlayer spacing, thus reducing the intercalation adsorption of PCE and improving the viscosity resistance of PCE [8].

## 4. Conclusions

In this study, we employed maltitol and CHPTAC as the raw materials to synthesize a new type of sacrificial agent which would adsorb onto Mt, and thus improving the clay tolerance of PCE. According to the experimental results, the optimal ratio of maltitol and CHPTAC is 1:6. Exceptionally, when KN-lm reaches 1.2 wt‰ in cement, the resulting cement slurry flow increases by 236% (1 h). Further investigations reveal that KN-lm would interact with Mt through electrostatic adsorption, and the sheet-like morphology was formed, which makes Mt no longer react with PCE. The XRD measurements do not show the intercalation behavior between KN-lm and Mt, and the mechanism of KN-lm and Mt was only electrostatic adsorption. Our results demonstrate that the addition of KN-lm could prevent PCE from intercalation adsorption with Mt. Our developed SA (KN-lm) possesses the merits of low-cost, environmentally friendly, and easy to be prepared, and therefore should be suitable for production.

## Figures and Tables

**Table 1:** Chemical composition and physical properties of cement and Mt.

Materials	Cement	Montmorillonite
Loss of ignition (wt%)	8.55	20.62
SiO_2_ (wt%)	26.28	53.77
AI_2_O_3_ (wt%)	8.77	15.07
Fe_2_O_3_ (wt%)	3.83	4.07
CaO (wt%)	44.92	4.04
MgO (wt%)	2.18	6.17
SO_3_ (wt%)	2.12	126
Density (g/cm^3^)	2.05	2.78
Specific surface area (m^2^/g)	932	377

**Table 2:** Fluidity contrast.

SA	w/c	Mt Content	PCE Content	Fluidity without SA	Fluidity with SA	Ref.
C14mim Br	0.35	1%	0.07%	150 mm	200 mm (0.05 wt% SA)	[8]
CTAB	0.35	1%	0.07%	150 mm	190 mm (0.05 wt% SA)	[21]
β-CD	0.29	1%	0.15%	170 mm	200 mm (0.1 wt% SA)	[20]
KN-lm	0.29	1%	0.3%	270 mm	320 mm (0.12 wt% SA)	This work

## Data Availability

The data presented in this study are available on request from the corresponding authors.
